# The Splitting of Fees

**Published:** 1911-09

**Authors:** 


### The Splitting of Fees

WHEN the Erie County Medical Society made public its report on the fee-splitting evil and the profession generally signed an agreement not to divide fees it made a pioneer move toward eradicating an evil of commercial medicine which was gratifying. At the same time, in making public in the daily press the findings of its committee and the wording of the report which lacked in spots that dignity of phraseology which is supposed to be the twin of science, it gave opportunity for jaundiced journalism to splash about in a sea of unrestricted words and hold the profession up to ridicule. That prophecy was made at the time the Buffalo newspapers were moralising on the decadence and commercialism of the profession which was so ulcerated of financial greed as to afflict its patients with unnecessary expense and decided hardship and make them the helpless victims of a high-grade form of graft. The prophecy made then has been fulfilled.

One of the cheaper magazines in its current issue has an article, lurid, reckless and ghastly in its yellowness, dealing with the question of fee splitting and the text of the article is the report of the Erie County Medical Society from which extracts are given which, in the absence of the portions preceding and following, are distinctly untempered. This is in no sense a complaint regarding the work of the society or its committee; it is merely a note of regret that any portion of the report should have been so worded that a cheap magazine might find fertile soil for the activities of its muckraking brigade. N. W. W.

